# Adam-Gibbs Formulation of Enthalpy Relaxation Near the Glass Transition

**DOI:** 10.6028/jres.102.015

**Published:** 1997

**Authors:** Ian M. Hodge

**Affiliations:** Imaging Research and Advanced Development, Eastman Kodak Company, Rochester, NY 14650-2116

**Keywords:** Adam-Gibbs, enthalpy relaxation, glass transition, nonlinear relaxation

## Abstract

The entropically based nonlinear Adam-Gibbs equation is discussed in the context of phenomenologies for nonlinear enthalpy relaxation within the glass transition temperature range. In many materials for which adequate data are available, the nonlinear Adam-Gibbs parameters are physically reasonable and agree with those obtained from linear relaxation data and thermodynamic extrapolations. Observed correlations between the traditional Tool-Narayanaswamy-Moynihan parameters are rationalized in terms of the Adam-Gibbs primary activation energy (Δ*μ*) determining how close the kinetic glass transition temperature can get to the thermodynamic Kauzmann temperature. It is shown that increased nonlinearity in the glass transition temperature range is associated with greater fragility in the liquid/rubber state above *T*_g_.

## 1. Introduction

Enthalpy relaxation near the glass transition is nonexponential and nonlinear. Nonexponential relaxation is very common in almost all types of condensed matter, but nonlinearity is unusual because for enthalpy relaxation it becomes significant at small departures from equilibrium (typically about 2 K in the temperature domain). Only nonlinear viscoelasticity in polymers is comparably important for such small and practically significant perturbations. In this article the phenomenology of nonlinear enthalpy relaxation near *T*_g_ is briefly reviewed, and nonlinearity is related to other commonly observed features of structural relaxation: the nonexponentiality just referred to, departures from Arrhenius behavior, and the relationship between kinetics and thermodynamics that is the subject of the article by Angell in this Special Issue [1].

Empirical evidence for nonlinearity is well established for volume relaxation (and thus of enthalpy as well since it includes volume). The most direct and compelling evidence is the asymmetric approach to equilibrium following temperature steps of opposite sign, reported by Hara and Suetoshi [2] for a soda-lime silicate glass (plotted in Ref. [3]) and by Kovacs for poly(vinyl acetate) [4] (presumably independently of Hara and Seutoshi given the inaccessibility of Ref. [2]). Another indication of nonlinearity was reported earlier by Lillie [5], who observed that the isothermal Newtonian viscosity of an unstabilized (nonequilibrium) inorganic glass changed with (annealing) time as the glass relaxed towards equilibrium. By taking the time dependence of the viscosity into account, Lillie’s shear stress relaxation data could be adequately represented by the Maxwell relation [3]

$$\sigma (t)=\sigma (0)\exp\left(\frac{-G_{\infty }t}{\eta_{0}(t)}\right),$$ (1)

where

*σ*: = shear stress,
*η*_0_: = Newtonian shear viscosity,
*G*_∞_: = limiting high-frequency shear modulus.

Since *G*_∞_ for inorganic glasses is a weak function of annealing, Eq. (1) implies a time-dependent shear stress relaxation time:

$$\tau (t)=\frac{\eta_{0}(t)}{G_{\infty }}.$$ (2)

Hopkins [6] observed that for thermorheologically simple materials, Eqs. (1) and (2) could be generalized to any nonexponential stress relaxation decay function, *ϕ* (*t*), using the reduced time *ζ* (*t*):

$$\zeta (t)\equiv \int_{0}^{t}\frac{dt^{\prime }}{\tau (t^{\prime })},$$ (3)

in which the zero of time is set when the material first falls out of equilibrium.^1^ The concept of the reduced time has a long history that, although not without interest, lies outside the scope of the present report. It should be mentioned, however, that it has been used by the mechanical engineering community for many years, who refer to it as an internal time or in terms of stress, strain, or material clocks. The need to incorporate and quantify nonlinearity in glassy state relaxation phenomenology was first recognized by Tool [7–9], whose work preceded the introduction of nonexponentiality and reduced time by several decades. Tool characterized the nonequilibrium state of a glass by introducing the fictive temperature, *T_f_*, defined as the temperature at which the excess nonequilibrium enthalpy (or any other property undergoing relaxation) would be the equilibrium value. Equilibrium is therefore characterized by *T_f_*(*t*) = *T*(*t*). The fictive temperature, and other aspects of the phenomenology of nonlinear relaxation, are discussed in detail in a recent review article [10] and in a book by Scherer [3]. The nonlinear enthalpy relaxation analog to Lillie’s time-dependent stress relaxation time is an enthalpic retardation time, *τ_H_*, that is a function of the time-dependent enthalpy:

$$H(t)=H_{0}+\Delta H\phi \{t,\tau_{H}[T(t),H(t)]\}.$$ (4)

Such nonlinear behavior can also be linearized using the reduced time, a technique first applied to structural relaxation by Gardon and Narayanaswamy [11]. For the ubiquitous stretched exponential form of the decay function. *ϕ* (*t*, *τ_H_*) in Eq. (4) is

$$\phi (t)=\exp(-\zeta {\left(t\right)}^{\beta }),\;1\geq \beta >0$$ (5)

where

*β*: = nonexponentiality parameter.

The nonlinearity of enthalpy relaxation has important practical implications. For example, the excess enthalpy (and entropy and volume) of a glass shortens the nonlinear relaxation and retardation times relative to the linear (equilibrium) values, resulting in appreciable relaxation (physical aging) in the glassy state. If relaxation in the glassy state was linear, annealing or physical aging would not occur on practically relevant time scales and the material engineering of glasses would be much simpler (and the science less interesting). The enthalpic retardation time is also a function of hydrostatic pressure and, for polymers, of viscoelastic stresses and strains (thus the use of stress and strain clocks referred to above). As a result, enthalpy relaxation is coupled to both the thermodynamic state of the glass and to viscoelastic retardation and relaxation processes. The nature of these couplings is complex and currently under study [12,13], but lies outside the present considerations and will not be discussed here in any detail. However, one important inference can be drawn from data on the effect of hydrostatic pressure on enthalpy and volume relaxation. Weitz and Wunderlich [14] measured the rates of enthalpy and volume relaxation in several pressure-densified glasses (PS, aPMMA, K/CaNO_3_, sucrose and phenolphthalein), formed by cooling through *T*_g_ under various hydrostatic pressures and then releasing the pressure. The glass densities at ambient pressure increased with cooling pressure (hence the name), and the excess enthalpy increased at pressures in excess of about 200 MPa. The physical aging rate for both enthalpy and volume increased with increasing density. Similar observations were reported by Prest and Roberts [15] for pressure-densified PVC, who found that the rate of enthalpy relaxation increased with cooling pressure. These results indicate unambiguously that excess (“free”) volume alone cannot be determining the retardation time of either volume or enthalpy in such glasses. However, an enthalpy- or entropy-dependent retardation time can readily accommodate the data because these quantities include the pressure-dependent internal energy as well as volume.

The most frequently used expression for *τ_H_*[*T*(*t*), *T*_f_(*t*)] in Eq. (4) is the Narayanaswamy equation [16] as modified by Moynihan [17] (henceforth referred to as NM):

$$\begin{array}{l} \;\tau_{H}[T(t),\;T_{f}(t)]\\ =A\;\exp\left(\frac{x\Delta h^{*}}{RT(t)}+\frac{(1-x)\Delta h^{*}}{RT_{f}(t)}\right)\;1\geq x>0, \end{array}$$ (6)

where

*A*: = preexponential constant,
*x*: = nonlinearity parameter,
Δh*: = effective activation energy just above *T*_g_.

**Fig. 1:**
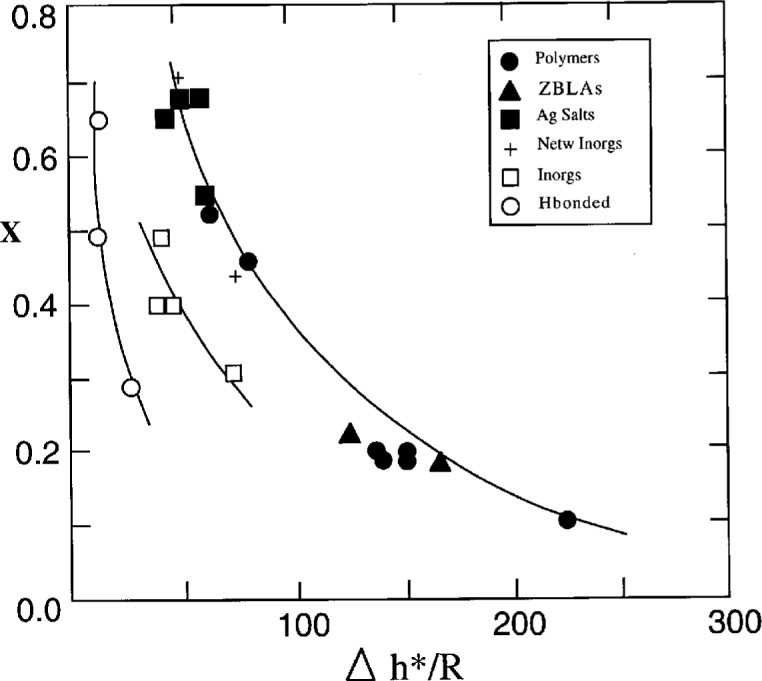
Plot of TNM parameters *x* vs Δh*. The lines are an aid to the eye, and do not conform to any specific mathematical expression.

**Fig. 2:**
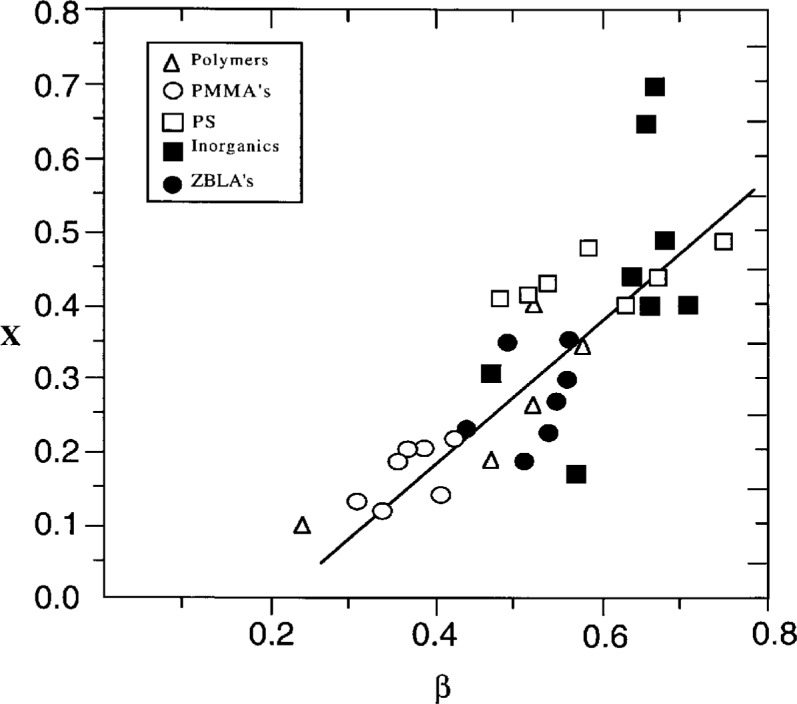
Plot of TNM parameters *x* vs *β*. The line is an aid to the eye, and is not a least squares fit.

The combination of Eqs. (3), (5) and (6) with Boltzmann superposition is the most frequently used nonlinear phenomenology for the study of enthalpy relaxation, and is referred to here as the Tool-Narayanaswamy-Moynihan (TNM) phenomenology. An equivalent but much less used expression for *τ_H_*(*t*) is the KAHR equation [18] (see Ref. [10] for details).^2^ The TNM parameters for about 30 materials have been collected in Ref. [10]. A striking feature of these data is the strong correlation between the parameters, exhibited in Fig. 1 in the form of *x* vs Δh*, and in Fig. 2 as *x* vs *β*. These correlations are somewhat tentative because the TNM parameters have large uncertainties compared with those of typical linear relaxation parameters, that arise in part because of the data analysis, and in part because the uncertainties themselves are correlated (the fitting parameters are not orthogonal in parameter search space). However, it can be confidently asserted that the uncertainties are not so large as to move a set of parameters from one end of the correlation range to the other, without producing fits that lie far outside experimental uncertainties in the heat capacity data, and the correlations will accordingly be accepted here at face value and as reflecting a physical reality demanding of explanation. Because of the empirical and phenomenological character of the TNM formalism, however, other expressions based on some sort of physical model are needed before any physical interpretation can be attempted of the parameter correlations, or of the parameters themselves. The nonlinear form of the entropic Adam-Gibbs (AG) equation is such an alternative, and is the focus of the present paper. In view of the finding that the TNM parameters sometimes vary with thermal history [10], however, the discussion and interpretations proffered here are restricted to relaxation within and very close to the glass transition temperature range. Parameters obtained solely from physical aging data are not discussed.

## 2. The Adam-Gibbs Equation

The original linear form of the Adam-Gibbs (AG) equation [19] derives from a generalization of the transition state expression

$$\tau_{H}(T)=A\;\exp\left(\frac{z^{*}\Delta \mu }{k_{B}T}\right),$$ (7)

where

Δ*μ*: = transition state activation energy,
z*: = temperature-dependent number of cooperatively rearranging molecular entities,
*k*_B_: = Boltzmann’s constant.

The temperature dependence of z* is determined by the macroscopic configurational entropy *S*_c_(*T*) [19]:

$$\frac{z^{*}(T)}{s_{c}^{*}}=\frac{N_{A}}{S_{c}(T)}.$$ (8)

where

*s*_c_*: = entropy of the smallest number of rearranging molecular entities,
*N*_A_: = Avogadro’s number.

Equations (7) and (8) yield

$$\tau_{H}(T)=A\;\exp\left(\frac{B}{TS_{c}(T)}\right),$$ (9)

in which

$$B=\frac{N_{A}s_{c}^{*}\Delta \mu }{k_{B}C},$$ (10)

where *C* = configurational heat capacity at *T*_2_.

In deriving these expressions the very weak temperature dependence of the preexponential factor, *A*, has been neglected^3^. Equations (7), (8), and (9) express the AG thesis that, analogous to the Gibbs-DiMarzio theory [20] for a thermodynamic glass transition, it is entropy that determines the rate of relaxation. The value of *S*_c_(*T*) is computed from

$$S_{c}(T)=\int_{T_{2}}^{T}\frac{\Delta C_{p}(T^{\prime })}{T^{\prime }}dT^{\prime },$$ (11)

where

Δ*C*_p_(*T*): = configurational heat capacity,
*T*_2_: = temperature at which the configurational entropy falls to zero.

The quantity Δ*C*_p_(*T*) is usually identified with the experimentally observed difference in liquid/rubber and glass/crystal heat capacities (but see later). The temperature *T*_2_ is conceptually identical with the thermodynamic Kauzmann temperature, *T*_K_ (see article by Angell [1]), but is distinguished from *T*_K_ here because for nonlinear enthalpy relaxation it is treated as a fitting parameter whose numerical equivalence with *T*_K_ is to be tested experimentally.

Nonlinear extensions of the AG equation have a history that goes back almost to the time of the original AG paper. Since these Symposium Proceedings are a celebration of 40 years of entropy, it is fitting that this history be given here, albeit briefly. Plazek and Magill [21] observed just one year after AG that, since the excess entropy of the glass is independent of temperature, the glassy state activation energy could be calculated from the parameter *B* obtained above *T*_g_. A ratio of glassy state to liquid activation energies of 0.338 was predicted, in excellent agreement with the experimental value of 0.333. Since this ratio equals the NM parameter *x* [Eq. (6)], it is evident that a link between nonlinearity and the AG prediction of Arrhenius behavior below *T*_g_ was implicit in the very earliest uses of AG. The connection was quantified 2 years later by Macedo and Napolitano [22], who deduced the temperature dependence of *S*_c_ needed to produce the VTF equation (see Ref. [1] and below) and found that the ratio of glassy to liquid activation energies was given by (1−*T*_2_/*T*_g_). This result was generalized by Howell et al. [23], who derived an expression for the ratio in terms of the fictive temperature dependence of *S*_c_:

$$\frac{E_{a}(\mathrm{glass})}{E_{a}(\text{liquid})}={\left(1+\left(\frac{d\ln S_{c}}{dT_{f}}\right)\right)}^{-1}.$$ (12)

Almost 10 years then elapsed before Scherer applied a form of the nonlinear AG equation to enthalpy relaxation for the first time [24]. He computed *S*_c_(*T*) from the experimentally determined temperature dependence of the configurational heat capacity, parameterized in the form Δ*C*_p_ = *a* − *bT*. Some time later, the present author used the hyperbolic form for Δ*C*_p_ (see below) to produce the simple nonlinear AG equations described next [25].

Nonlinear generalizations of the AG equation are obtained by making *S*_c_ a function of the fictive temperature, rather than the thermodynamic temperature:

$$\tau_{H}(T,T_{f})=A\;\exp\left(\frac{B}{TS_{c}(T_{f})}\right).$$ (13)

The specific form of *τ_H_*(*T*, *T*_f_) is determined by the temperature dependence of Δ*C*_p_. For many inorganic glasses, this is well approximated by the hyperbolic expression

$$\Delta C_{p}(T)=\frac{CT_{2}}{T}.$$ (14)

The temperature dependence of Δ*C*_p_ for polymers is weaker, lying between the hyperbolic dependence and constancy, but the results discussed here are not particularly sensitive to the form of Δ*C*_p_(*T*) and Eq. (14) will be used throughout the present discussion. Insertion of Eq. (14) into Eqs. (11) and (13) yields [10, 25–27]

$$\tau_{H}(T,T_{f})=A\;\exp\left(\frac{B}{T(1-T_{2}/T_{f})}\right).$$ (15)

Equation (15) is referred to here simply as the nonlinear AG equation, despite the objection that other nonlinear AG equations result from different functional forms for Δ*C*_p_(*T*) (see below). This nomenclature is countenanced because of the convenient fact that the linear from of Eq. (15), obtained by placing *T*_f_ = *T*, is the celebrated Vogel-Tammann-Fulcher (VTF) equation [28]^4^

$$\tau_{0}(T)=A\;\exp\left(\frac{B}{T-T_{0}}\right),$$ (16)

where the nonlinear enthalpic *T*_2_ and linear VTF *T*_0_ parameters are physically equivalent but are distinguished here because, as with the relation between *T*_2_ and *T*_K_, it is a matter for experiment to decide whether they are numerically equal. An example of an alternative nonlinear AG equation is that obtained by assuming that Δ*C*_p_ is independent of temperature:

$$\tau_{0}(T,T_{f})=A\;\exp\left(\frac{B}{T\;\ln(T_{f}/T_{2})}\right).$$ (17)

Over the narrow temperature ranges near *T*_g_ that are considered here, Eq. (17) gives fits to experimental data that are indistinguishable from Eq. (15), albeit with different *B* and *T*_2_ parameters. Indeed, the ranges in temperature are small enough that the nonlinear AG and NM equations also give equally good fits. Relationships between the NM and AG parameters are readily derived by equating the activation energies above and below *T*_g_ and applying the approximation *T*_f_ ≈ *T* ≈ *T*_g_:

$$\Delta h^{*}=\left(\frac{d\;\ln\;\tau_{H}(T)}{d(1/T)}\right),$$ (18)

and

$$x\Delta h^{*}=\left(\frac{\partial \;\ln\;\tau_{H}(T,T_{f})}{\partial (1/T)}\right)T_{f}.$$ (19)

For Eqs. (6) and (15), this procedure yields [10, 25–27]

$$x\approx -T_{2}/{T^{\prime }}_{f}\approx -T_{2}/T_{g}$$ (20)

and

$$\frac{\Delta h^{*}}{R}\approx \frac{B}{x^{2}}\approx \frac{B}{{\left(1-T_{2}/T_{g}\right)}^{2}},$$ (21)

where *T*_f_′ is the glassy state value of *T*_f_, obtained after cooling through *T*_g_ but before any significant annealing or physical aging can occur.^5^ Equations (20) and (21) are found to be accurate when the same enthalpy relaxation data are analyzed using the NM and AG equations [10], so that AG parameters can be confidently estimated from the more widely published NM parameters.

## 3. Physical Interpretation of Nonlinearity Parameters

The nonlinear AG equation has physically meaningful parameters that allows several questions to be addressed:

- The validity of Eq. (15) can be tested by comparing the best fit nonlinear enthalpic values of *T*_2_ with available Kauzmann temperatures *T*_K_.
- Equation (20) reveals that the NM nonlinearity parameter, *x*, is determined by how close the kinetic *T*_g_ can get to the thermodynamically defined temperature of zero excess entropy, *T*_2_. This raises the question of what determines the ratio *T*_g_/*T*_2_.
- Equation (10) indicates that Δ*μ* is calculable from experimental values of the AG parameter *B*, if Δ*C*_p_(*T*_2_) and *S*_c_* are known or can be estimated.
- Equations (8), (10) and (14) indicate that z* at *T*_g_ is given by
  $$z^{*}(T_{g})=\frac{s_{c}^{*}N_{A}}{S_{c}(T_{g})}=\frac{R\ln(W^{*})}{C(1-T_{2}/T_{g})}=\frac{R\ln(W^{*})}{Cx},$$ (22)
  where W*(≥ 2) = minimum number of configurations needed for cooperative relaxation. Since large values of z*(*T*_g_) imply increased cooperativity, it is of interest to compare it with some independent measure of cooperativity.

### 3.1 Values of *T*_2_, *T*_0_ and *T*_K_

Table 1 summarizes the values of *T*_2_ and *T*_K_ for the handful of materials for which both are reliably known, together with VTF values of *T*_0_ obtained from linear dielectric and viscoelastic relaxation data above *T*_g_. For most materials there is extremely good agreement between *T*_2_ and *T*_K_ (well within uncertainties), but there is a significant discrepancy for PS. There is also good agreement between the nonlinear *T*_2_ and linear *T*_0_ values, except for PS again. Given the limited number and variety of materials it is inappropriate to draw firm conclusions from these data, but it is worth noting that the best agreement between *T*_2_ and *T*_K_ is found for B_2_O_3_ and As_2_Se_3_, for which the values of *T*_K_ are most reliable and for which the temperature dependence of Δ*C*_p_ is very close to the hyperbolic form of Eq. (14). For glycerol, there is additional excellent agreement between the nonlinear AG value of *T*_2_ and the value of *T*_2_ obtained from ac calorimetry [30] in which enthalpy relaxation is determined in the linear regime of small (sinusoidal) temperature perturbations. These observations encourage the belief that nonlinearity is indeed determined by the ratio of the kinetically determined *T*_g_ and the thermodynamic Kauzmann temperature *T*_K_. The notable exception to this agreement is PS. This disagreement cannot be ascribed to experimental uncertainty, because PS has the most reliable value for *T*_K_ of any polymer, and its enthalpy relaxation parameters have been characterized by more independent groups than for any other material and found to be in very good agreement [10]. A possible reason for *T*_2_ being so far below *T*_K_ for PS is that Δ*C*_p_ is not entirely configurational, as suggested by Goldstein for glasses in general [31, 32].^6^ If *T*_K_ is forced to equal *T*_2_ then only about 40 % of Δ*C*_p_ is configurational, a figure that lies at the low end of the range estimated by Goldstein. This interpretation is very speculative, however, and should be regarded more as a logical and physical possibility than as a compelling explanation.

### 3.2 Values of Δ*μ*

Values of the AG “primary” activation energy, Δ*μ*, can be computed from Eq. (10) once *S*_c_* and Δ*C*_p_ are known, although the reliability of such calculations is subject to a number of caveats. First, several types of molecular entities are involved in a typical cooperative relaxation event, and derived values of Δ*μ* are some sort of average that cannot be expected to have anything more than a semiquantitative significance. Second, Δ*C*_p_ is an extensive variable and the unit of mass must be specified. An appropriate unit for polymers is the Wunderlich bead [33], which can be identified with segmental units whose relative rotation is a natural candidate for the primary molecular motion. The AG activation energy Δ*μ* for polymers can then be identified with intersegmental rotational energy barriers. The question of mass is more problematic for nonpolymeric organic and inorganic glasses, and apart from a few brief references to literature data the analysis given here is restricted to polymers. Finally, a value for s_c_* must be specified. This has usually been taken to be *k*_B_ ln 2, since there must be a minimum of two configurations available for relaxation to occur (those before and after rearrangement). For polymers, which are geometrically constrained to essentially one dimension, it has been argued [10, 25–27] that *S*_c_* is better approximated as *k*_B_ ln 2^3^. This value is based on the assumptions that a minimum of three segments must cooperatively rearrange for the classic crankshaft motion to occur, and that there are two nonequivalent rotamers per bond. This estimate of *S*_c_* is uncertain, but is clearly better than *k*_B_ ln 2 and, given the unknown contribution to rotational barriers by interchain interactions and the fact that several types of segmental pairs must be averaged for a typical polymer, is considered adequate for the present discussion. Values of Δ*μ*/*k*_B_ per bead are summarized in Table 2 for four polymers, together with the number of beads and values of the number of cooperatively rearranging beads at *T*_g_ (z*, to be discussed later).

The values Δ*μ* calculated for polymers assuming *S*_c_* = *k*_B_ ln 2 are consistent with expected intersegmental rotational energy barriers, as modified by interchain interactions. The value for PS is rather high, but might be associated in some way with constrained motion of the bulky phenyl ring as intersegmental rotation occurs. The value for Δ*μ* is also physically reasonable for inorganic silicates, being comparable with the Si – O bond energy if *S*_c_* = k_B_ ln 2 is assumed [24]. For a series of lead phosphate and lead/iron phosphate glasses, Sales [34] determined the product Δ*μS*_c_* and obtained values of *S*_c_* by assuming that Δ*μ* equaled the *P*–O bond energy. For the lead phosphates he obtained a physically sensible value for W* = exp(*S*_c_*/*k*_B_) of 4.6 that was independent of lead content. This value increased to 24.5 when 25 % of the lead was replaced with ferric iron, consistent with a more cooperative motion being required as the geometric constraints caused by the (presumably six fold) crystal field stabilized coordination of the iron increased.

### 3.3 Values of z* at *T*_g_

**Fig. 3:**
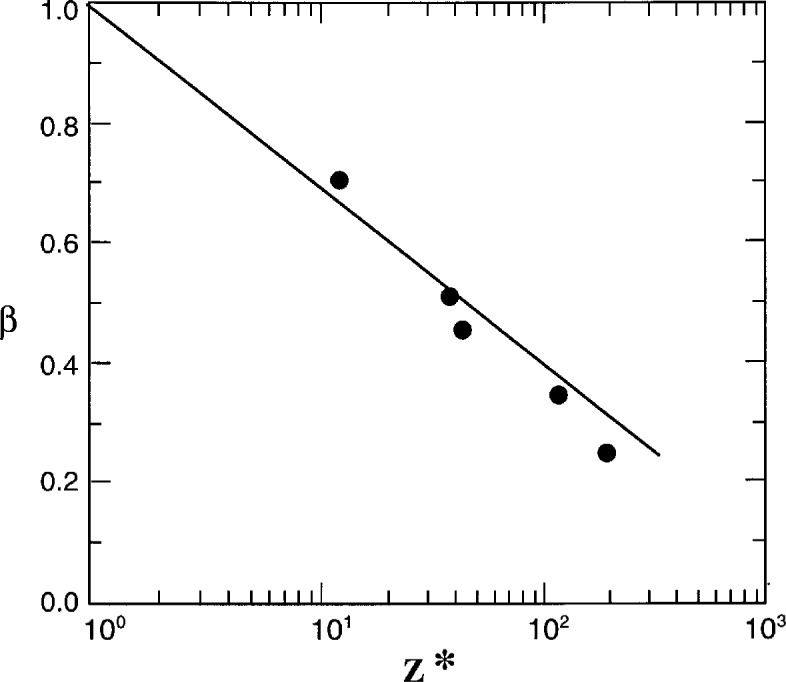
Plot of nonexponentiality parameter *β* vs Adam-Gibbs quantity z*. The line is an aid to the eye, to suggest that *β* → 1 as z* → 1, and is not a least squares fit.

Values of the number of polymer beads that cooperatively rearrange at *T*_g_ are included in Table 2, and are physically reasonable with the exception once again of PS. There is a clear correlation between z*(*T*_g_) and the stretched exponential parameter *β*, which is exhibited in Fig. 3 in the form of a plot of *β* vs log z*. The logarithmic scale for z* has no theoretical basis, and is chosen simply because it conveniently produces a near linear relation. Note that the decay function becomes exponential (*β* = 1) as z* approaches unity, consistent with relaxation being (by definition) no longer cooperative when only a single molecular entity is involved. The other expected limit, that the relaxation spectrum becomes infinitely broad (*β* → 0) as z* → ∞, is not evident although the apparent limit of z* ~ 2000 as *β* → 0 is large enough to be considered macroscopic and physically indistinguishable from mathematical infinity. In any event, the clean correlation between z* and *β* is in accord with physical expectations, and provides further evidence that the nonlinear AG parameters are physically reasonable.

### 3.4 The Ratio *T*_g_/*T*_2_ and Parameter Correlations

**Fig. 4:**
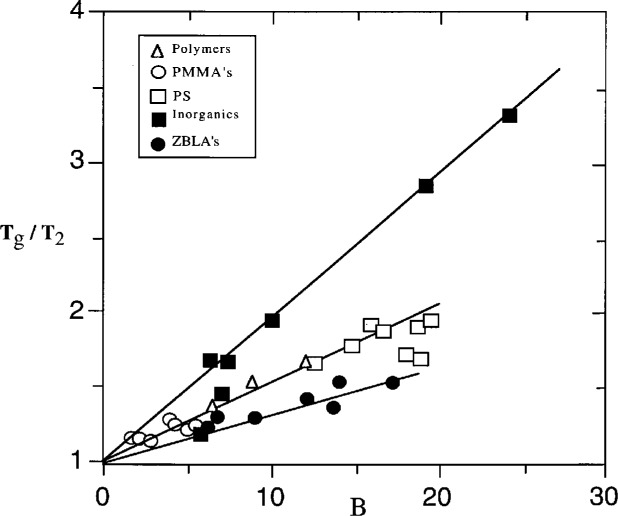
Plot of *T*_g_/*T*_2_ ≈ (1 − *x*)^−1^ vs *B* ≈ *x*^2^Δh*. The lines are an aid to the eye, to suggest that *T*_g_ → *T*_2_ as *B* → 0 for all material types, and are not least squares fits.

In seeking the factor(s) that determine how close *T*_g_ can get to *T*_2_, and therefore the degree of nonlinearity, it is natural to consider the primary activation energy, Δ*μ*. The hypothesis to be tested is that lower values of Δ*μ* allow *T*_g_ to more closely approach *T*_2_. This can be assessed by plotting *T*_g_/*T*_2_ ≈ (1 − *x*)^−1^ as a function of *B* ≈ *x*^2^Δh*. Although the proportionality factor between *B* and Δ*μ* contains the material dependent ratio *S*_c_*/Δ*C*_p_ [Eq. (10)], this ratio cannot be zero and the limit *B* → 0 corresponds uniquely to Δ*μ* → 0. A plot of *T*_g_/*T*_2_ ≈ (1 − *x*)^−1^ vs *B* ≈ *x*^2^Δh* is shown in Fig. 4, from which it is clearly apparent that *T*_g_/*T*_2_ does indeed unambiguously extrapolate to unit as *B* approaches zero. The AG-based hypothesis that Δ*μ* determines *T*_g_/*T*_2_, and the associated proportionality between *B* and (*T*_g_/*T*_2_ − 1), correspond to the inverse correlation between *x* Δh* seen in Fig. 1. This correspondence is readily demonstrated once it is recognized that, by binomial expansion, the quantity (*T*_g_/*T*_2_ − 1) ≈ (1 – *x*)^−1^ − 1 is approximately equal to (1 − *T*_2_/*T*_g_) ≈ (accurate within the typical experimental uncertainty of 10 % in (1 − *x*)^−1^ for 0 > *x* > 0.5). There is a suggestion in Fig. 4 that different material types fall on different correlation lines, although the uncertainties in the parameters make this observation less than compelling. Because of these uncertainties, and the ambiguity in choosing the appropriate unit of mass, no attempt is made here to correlate either experimental values of Δ*C*_p_ or estimates of *S*_c_* with the possible different slopes.

**Fig. 5:**
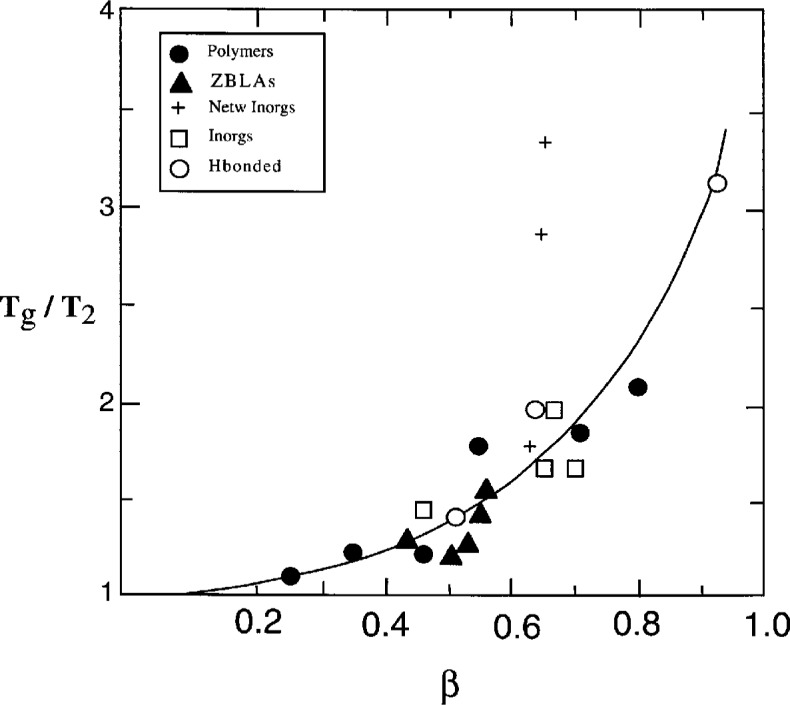
Plot of *T*_g_/*T*_2_ ≈ (1 − *x*)^−1^ vs *β*. The line is an aid to the eye, to suggest that *β* → 0 as *T*_g_ → *T*_2_, and is not a least squares fit.

The correlation between the stretched exponential parameter *β* and *x* (Fig. 2) can also be rationalized in terms of *T*_g_/*T*_2_, if the ancillary assumption is made that the large values of z* associated with small values of *T*_g_/*T*_2_ correspond to an increased degree of cooperativity (as suggested by the polymer data plotted in Fig. 3), and that low values of *β* are associated with such cooperativity. A plot of *β* versus *T*_g_/*T*_2_ is shown in Fig. 5 for all materials for which data are available and, except for the network silicate glasses, the correlation is good and consistent with the physically reasonable expectation (see Sec. 3.3) that *β* → 0 as *T*_g_/*T*_2_ → 1 (a similar trend is suggested by some linear dielectric relaxation data [35], that are much less uncertain than the nonlinear data under discussion here).

## 4. Summary

The entropy based nonlinear Adam-Gibbs account of enthalpy relaxation within the glass transition temperature range provides a consistent account of the correlations observed between the TNM relaxation parameters, with physically reasonable parameters in most cases. Such uniform consistency is perhaps unexpected, since the Adam-Gibbs concept of cooperatively relaxing regions, and the assumption that the size of such regions is determined by the macroscopic configurational entropy, must both be considered more as heuristic aids than as rigorous theoretical concepts. The central AG results, that nonlinearity is associated with how close the kinetic glass temperature *T*_g_ is to the thermodynamic Kauzmann temperature *T*_K_, and that lower values of *T*_g_/*T*_K_ can be identified with increased fragility [36] as proposed by Angell [37], suggest that increased nonlinearity should be regarded as a hallmark of greater fragility. The correlations between the various nonlinear enthalpy relaxation parameters can then be regarded as having the same physical cause as those that form the basis for the strong/fragile classification scheme for linear relaxation phenomena near but above *T*_g_.

## Figures and Tables

**Table 1:** Values of nonlinear AG *T*_2_, linear VTF *T*_0_, and thermodynamic *T*_K_^a^

Material	*T*_2_ (K)	*T*_0_ (K)	*T*_k_ (K)
PVAc	225	238 (ε*)^b^	
		247 (WLF)^c^	
aPMMA	325	301 (WLF)	335^d^
		222 (ε*)	
PS	210	323 (WLF)	270
BPAPC	325	385 (ε*)	
B_2_O_3_	321		335
As_2_Se_3_	237		236
Glycerol	134 (128 linear)^e^	132 (ε*)	135

a See Ref. [10] for original references to individual values of *T*_2_, *T*_0_ and *T*_K_.

b Obtained from fitting dielectric data to the VTF equation [Eq. (16)].

c From the WLF parameters *C*_2_^g^ obtained from fits to viscoelastic shift factors, using *T*_0_ = *T*_g_ – *C*_2_^g^. For references to the original data, see J. D. Ferry, Viscoelastic Properties of Polymers, Third Edition, John Wiley and Sons (1980).

d The value of *T*_K_ for uncrystallizable atactic PMMA was estimated from the value for crystalline syndiotactic PPMA, using the approximation that *S*_c_(*T*_g_) is the same for all tacticities and the experimental fact that Δ*C*_p_(*T*) is identical for all tacticities of PMMA [29], so that *T*_g_ − *T*_K_ is independent of tacticity.

e From ac calorimetry data (Ref. [30]).

**Table 2:** Values of Δ*μ*/*k*_B_(*k*K per bead) for polymers obtained from nonlinear B parameter

Polymer	Number of beads^a^	Δ*μ*/*k*_B_ for W*		z*(*T*_g_) (beads)
ln 2	ln2^3^
PVAc	4	6.8	2.3	40
PS	3	18	6.0	13
aPMMA	3	4.9	1.6	120
	4	3.7	1.2	
BPAPC	5	9.8	3.3	45
	6	8.2	2.7	

a Number of beads per monomer unit.

## Footnotes

1 Starting the reduced time “clock” at a later time can produce artifacts arising from the memory effect associated with nonexponential relaxations, in which relaxation from a particular state is determined not only by what that state is, but also on how it was reached. For a detailed discussion, see Refs. [10] and [13].

2 The historical reason for the KAHR formalism not often being used is that it is cast in the form of coupled nonlinear partial differential equations, whose solution requires more computing time than does Boltzmann integration of responses linearized using the reduced time.

3 The preexponential factor, *A*, is regarded here to be just as empirical as the arbitrary reference temperatures that often replace it in other equations in the literature (such as the WLF equation). This position is particularly appropriate for the AG equations, since *A c*orresponds to the relaxation time in the limit of high temperature, where the AG concept of cooperatively rearranging groups can be expected to break down. Accordingly, the same symbol for the preexponential factor is used in all the equations presented here, despite the fact that its numerical value differs for different equations (sometimes substantially).

4 The derivation of the VTF equation from inserting the hyberbolic form for Δ*C*_p_(*T*) into the entropic AG expression (9) has a history that also dates back almost to the origin of AG. References are given in Ref. [10], but the detailed history is left for another place and time.

5 This value of *T*′_f_ is the definition of choice for *T*_g_.

6 More precisely, relaxation may be determined by only some of the degrees of freedom that contribute to Δ*C*_p_. It is also possible that only a part of the entropy of fusion is configurational (or determines relaxation), in which case values of *T*_K_ obtained by assuming that 100 % of the entropy of fusion is configurational would be too low, and the difference between *T*_2_ and *T*_K_ would be even larger. If the configurational fraction of the heat of fusion is the same as the configurational fraction of Δ*C*_p_, however, then the original values of *T*_K_ are clearly valid.
